# Dr. Smelfungus

**Published:** 1853-01

**Authors:** 


*Dr. Smelfungus.* — We tender our thanks to the editor of the Philadelphia Medical Journal, for his complimentary notice of the Buffalo Medical Journal, and the flattering compliment which he pays to our editorial labors. We cannot, however, assume the authorship of the Smelfungus papers. There are by a talented correspondent from whom we shall always be happy to receive communications, and for whom we predict a high reputation as a medical writer.

